# Integrated analysis of DNA-methylation and gene expression using high-dimensional penalized regression: a cohort study on bone mineral density in postmenopausal women

**DOI:** 10.1186/s12920-018-0341-2

**Published:** 2018-03-07

**Authors:** Tonje G. Lien, Ørnulf Borgan, Sjur Reppe, Kaare Gautvik, Ingrid Kristine Glad

**Affiliations:** 1 University of Oslo, Department of Mathematics, P.O Box 1053, Oslo, 0316 Norway; 2 Oslo University Hospital, Department of Medical Biochemistry, Oslo, Norway; 3 Lovisenberg Diakonale Hospital, Unger-Vetlesen Institute, Oslo, Norway; 4 University of Oslo, Institute of Basic Medical Sciences, Oslo, Norway

**Keywords:** Bone, DNA-methylation, Gene expression, Individual penalties, Integration, Lasso, Ridge regression

## Abstract

**Background:**

Using high-dimensional penalized regression we studied genome-wide DNA-methylation in bone biopsies of 80 postmenopausal women in relation to their bone mineral density (BMD). The women showed BMD varying from severely osteoporotic to normal. Global gene expression data from the same individuals was available, and since DNA-methylation often affects gene expression, the overall aim of this paper was to include both of these omics data sets into an integrated analysis.

**Methods:**

The classical penalized regression uses one penalty, but we incorporated individual penalties for each of the DNA-methylation sites. These individual penalties were guided by the strength of association between DNA-methylations and gene transcript levels. DNA-methylations that were highly associated to one or more transcripts got lower penalties and were therefore favored compared to DNA-methylations showing less association to expression. Because of the complex pathways and interactions among genes, we investigated both the association between DNA-methylations and their corresponding cis gene, as well as the association between DNA-methylations and trans-located genes. Two integrating penalized methods were used: first, an adaptive group-regularized ridge regression, and secondly, variable selection was performed through a modified version of the weighted lasso.

**Results:**

When information from gene expressions was integrated, predictive performance was considerably improved, in terms of predictive mean square error, compared to classical penalized regression without data integration. We found a 14.7*%* improvement in the ridge regression case and a 17% improvement for the lasso case. Our version of the weighted lasso with data integration found a list of 22 interesting methylation sites. Several corresponded to genes that are known to be important in bone formation. Using BMD as response and these 22 methylation sites as covariates, least square regression analyses resulted in *R*^2^=0.726, comparable to an average *R*^2^=0.438 for 10000 randomly selected groups of DNA-methylations with group size 22.

**Conclusions:**

Two recent types of penalized regression methods were adapted to integrate DNA-methylation and their association to gene expression in the analysis of bone mineral density. In both cases predictions clearly benefit from including the additional information on gene expressions.

**Electronic supplementary material:**

The online version of this article (10.1186/s12920-018-0341-2) contains supplementary material, which is available to authorized users.

## Background

Poor bone health and low bone mineral density (BMD) lead to reduction in bone mechanical strength. BMD is commonly used in clinical practice as a surrogate measure of bone strength. Osteoporosis (OP) is diagnosed when the bone mineral density is more than 2.5 standard deviations below that of a young adult (30–40 years old), healthy women reference population [1]. In the Western world, more than 40% of women over 50 years experience OP and low energy fractures, and the highest rates are found in the Scandinavian countries [2].

The number of publications describing DNA-methylation in bone cells is limited, but the understanding of its importance is rapidly increasing. DNA-methylation is an important factor in the development and function of bone cells, and therefore also in skeletal disease characteristics [3, 4]. In this paper we explore the impact of genome-wide DNA-methylations on BMD further.

In the present study of 80 postmenopausal women (50–84 years), we have measured both global DNA-methylation and global RNA transcripts obtained from the same bone biopsies. Combining this information can give us additional strength in the functional analysis of DNA-methylation. The relationship between DNA-methylation and gene expression is complex [5–7]. While increased methylation in promoter regions tends to reduce transcription, probably by inhibition of transcription factor binding, increased methylation within the transcribed part of DNA often increases transcription, probably due to reduced use of spurious intergenic promoters [8]. Also, binding of transcription factors to a promoter region may promote or inhibit DNA-methylation depending on the properties of that factor. In addition, cellular functions are largely influenced by networks and complex pathways involving several genes, also on other chromosomes, which again have both positive and negative influence.

This paper aims to identify genome-wide DNA-methylation sites explaining the variation in BMD in postmenopausal women, while integrating the association between each CpG site and gene expression using a common set of bone biopsies. We assume that methylations strongly associated with transcripts are most relevant in regulation of bone mineral density. This assumption is based on results from several recent studies: We earlier studied bone DNA methylations in the 100 genes representing transcripts most significantly associated with BMD [9]. These DNA methylations were significantly correlated to several of the 100 transcripts. We have also showed, that methylation of the bone SOST promoter is associated with reduced level of its protein sclerostin, an important bone anabolic inhibitor [10]. Furthermore, Del Real et al. [11] performed global DNA methylation and transcription analysis of human mesenchymal stem cells (hMSCs), the precursors of osteoblasts, from femoral heads of women undergoing hip replacement due to fractures using controls with hip osteoarthritis. The authors identified differentially methylated loci situated in genomic regions with enhancer activity, being associated with differentially expressed genes/transcript levels enriched in pathways related to hMSC growth and osteoblast differentiation.

We fit a multivariate regression model using BMD as response and genome-wide DNA-methylations as covariates. The number of covariates is much greater than the number of individuals, so ordinary linear regression does not give a unique solution, and it follows that penalized regression methods, also known as shrinkage or regularization methods, are needed. We include gene expression data in the analysis, by allowing for individual penalties in the regression, and let the gene transcript levels guide these penalties. Both cis (DNA-methylation and transcript from the same gene) and trans (DNA-methylation and transcript from different genes) associations are investigated. We point out that we study associations, and that such effects may be causal, but do not necessary need to be. Only in the case when a change in one variable causes a corresponding change in the other variable, can the effect be called causal.

One of the most used penalized regression methods is ridge regression [12]. This method does not assume the quite strong assumption of sparsity, in the sense that only a fairly small number of covariates has an effect on the response variable. It follows that ridge regression includes all covariates in the final model. A ridge regression method which allows for individual penalties is the adaptive group-regularized ridge regression [13]. This is a generic method, which allows for ranked penalties for groups of covariates. We used this method by dividing the methylation sites into groups based on their association to gene transcript levels. We show that this method improves the predictive ability compared to the classical ridge regression in which the external information from gene expressions is not included.

Typically, two aspects are important when fitting a regression model. First a model should be able to predict future data, secondly it should be interpretable [14]. A large number of covariates may complicate the interpretation, especially when the number of covariates *p* is larger than the number of individuals *n*. By doing dimension reduction we can get a shortlist of the most important variables. Using this approach, assuming sparsity, we can use the highly popular lasso method [15]. For our dataset it does not give as good prediction as ridge regression, but the resulting short list of genes is easier to interpret. Within this lasso framework, the weighted lasso with data integration allows for individual penalties based on external information [16]. We extend this method further to suit our particular data types. Using this modified method, we improve predictive performance, compared to the classical lasso, where gene expression is not integrated.

This paper is organized as follows: In the method section we first present an overview of the data, next in the section “Quantifying the strength of association between DNA-methylation and gene expression” we present how we test for a significant association between each DNA-methylation and the gene transcript levels, and how we find the *p*-values adjusted for multiple testing. The adjusted *p*-values are our measure of association between DNA-methylation and gene expression. In the section “Penalized regression using penalty multipliers”, the essential concept of individual penalties in penalized regression is presented. In our penalized regression, with bone mineral density as response, each covariate (hence DNA-methylation) gets an individual penalty. The relationship between these individual penalties and the adjusted *p*-values are described in the section “Mapping the penalty multipliers to the adjusted *p*-values”. Lastly, in the sections “Adaptive group-regularized ridge regression” and “Extensions to the weighted lasso with data integration”, we present the details in the generic adaptive group-regularized ridge regression method [13], and the extensions to weighted lasso with data integration to fit to our integration setting. The functional enrichment analysis is described in the section “Functional enrichment analysis”. Section “Results” presents the results from the two methods and their consensus, and lastly we discuss in the section “Discussion”, the methodology and the biological findings in this study.

## Methods

### Cohort description

Postmenopausal ethnic Norwegian women were consecutively recruited at Lovisenberg Diakonale Hospital, the Out-patient Clinic, in Oslo. The survey was performed in 2004–2010. The women showed BMD varying from severely osteoporotic to normal and did not have other diseases or medication known to affect the skeleton. Extensive clinical and biochemical information on this cohort are available in [17].

**Fig. 1:**
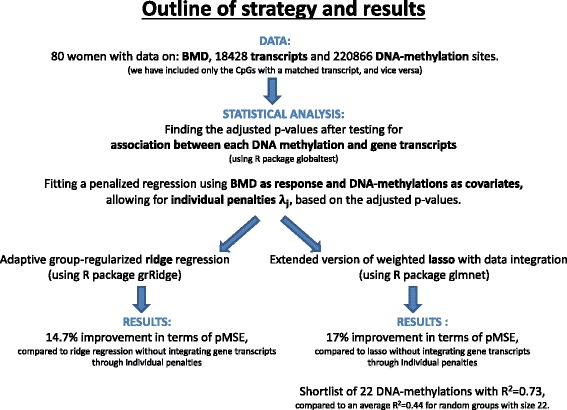
Outline of strategy and results. An overview of the statistical methods and the following results for each of the two methods adaptive group-regularized ridge regression and our version of the weighted lasso with data integration. The results are presented by change in predictive mean square error (pMSE) as described in the section “Results”, when comparing these two methods to ridge regression and lasso, respectively, thus not integrating gene expression data

Trans-ilical bone biopsies were obtained in 80 women, the largest cohort of today, and global transcripts and DNA-methylation levels were measured from the same samples. Total RNA was subjected to analysis on HG-U133 plus 2.0 chips (Affymetrix, Santa Clara, CA), and 18428 transcripts were studied after filtering those expressed at low levels. For more details, see [17]. DNA-methylation mapping was performed using the Illumina Infinium HumanMethylation450 BeadChip, which is designed to provide coverage throughout gene regions, as shown in Additional file 1: Figure S5. To determine the quantitative measurement of methylation for each CpG (beta values), the data was prepared in BeadStudio (Illumina). Quality checks were followed by BeadStudio and minfi [18], together with preprocessing, and lastly normalization by a beta-mixture quantile normalization method (BMIQ), which is used to correct for probe design bias in Illumina Infinium 450k DNA methylation data [19]. Based on gene symbols from Illumina, we match methylation sites and genes, and use only those methylation sites where we find a match in our dataset of transcripts, leaving us with a total of 220866 DNA-methylation sites. Next follows a detailed description of the statistical methods, as shown in the flowchart in Fig. 1.

### Quantifying the strength of association between DNA-methylation and gene expression

When investigating the association between each DNA-methylation and gene transcripts, we look at two scenarios, first each DNA-methylation against all possible gene transcripts in the analysis, and secondly each DNA-methylation against only the cis related genes. In both cases, we quantify the strength of an association by using the utility test called the “global test” [20]. The null hypothesis is that there’s no association between gene expressions and DNA-methylation, and the alternative hypothesis is that one or more gene transcript levels are associated with the DNA-methylation level. This test is valid for both high-dimensional data (*p*>*n*) as well as the case where there are more individuals than covariates. The association between DNA-methylations and gene expressions can be both positive and negative. See Additional file 2 for more details. We performed tests for each DNA-methylation, and got a corresponding *p*-value *p*_*j*_ for *j*=1,…,*p*. Since a large number of tests is performed, we adjust for multiple testing by controlling the false discovery rate (FDR) [21]. We use the adjusted *p*-value, called the *q*-value *q*_*j*_, as a measure of the strength of the association between the particular DNA-methylation and the gene expressions. Moreover, we interpret low values of *q*_*j*_, as a strong association.

### Penalized regression using penalty multipliers

We fit a multivariate linear regression, using BMD as response and DNA-methylations as covariates. For each individual *i*, the BMD is noted *y*_*i*_ and DNA-methylation **x**_*i*_=(*x*_*i*1_,…,*x*_*ip*_)^*T*^, where *i*=1,…,*n*. Without loss of generality, let the data be centered such that the intercept in the regression is equal to zero. Since we have more covariates than observations, we will use penalized regression. The regression coefficients ***β***=(*β*_1_,…,*β*_*p*_)^*T*^ are then estimated by minimizing the residual sum of squares together with a restriction on the coefficients $ \sum _{j=1}^{p} J\left (\beta _{j} \right) \leq s $. In ridge regression $J\left (\beta _{j} \right) = \beta _{j}^{2}$ and in the lasso *J*(*β*_*j*_)=|*β*_*j*_|. This optimization is equivalent to minimizing the penalized sum of squares

1 $$ \sum_{i=1}^{80} \left(y_{i} - \mathbf{x}_{i}^{T} \boldsymbol{\beta}\right)^{2} + \lambda \sum_{j=1}^{p} J\left(\beta_{j}\right),  $$

over all ***β***, using one common penalty parameter *λ*. Alternatively, one can allow for multiple penalties, as described in several papers [22–25]. When allowing for individual penalties per covariate we replace the penalty term $\lambda \sum _{j=1}^{p} J\left (\beta _{j}\right)$ in (1) by $ \sum _{j=1}^{p} \lambda _{j} J\left (\beta _{j}\right)$. The larger the difference between the smallest and largest *λ*_*j*_, the more favored certain covariates become. We write

2 $$\begin{array}{*{20}l} \lambda_{j} &= \lambda \cdot l_{j}, \end{array} $$

where *l*_*j*_>0 is a penalty multiplier and will be defined in the next section. Note that it is possible, and sometimes desirable, to have equal *λ*_*j*_ within a group of covariates. The optimization of the weighted penalty regression can, after some scaling of the data, be rewritten as the optimization of the classical penalty regression without multiple penalty terms. For more details, see Additional file 2: Additional materials.

### Mapping the penalty multipliers to the adjusted *p*-values

To link the adjusted *p*-values *q*_*j*_, described in the section “Quantifying the strength of association between DNA-methylation and gene expression”, with the penalty multiplier *l*_*j*_ we need some function on the form *l*_*j*_=*h*(*q*_*j*_). The general idea for such a function is that it must reflect the possible minimum and maximum penalty weight, and the relative penalty between the covariates. A simple example is

$$h\left(q_{j}\right)= \begin{array}{ll} 1, & q_{j} < c \\ \infty, & q_{j} \geq c, \end{array} $$

for some threshold *c* which reflects the significance level. This is the same as excluding the non-significant covariates from the analysis. We use two more appropriate and sophisticated versions of *h*(·), which do not exclude any of the covariates from the initial analysis. For the function to be meaningful it should

- be a monotone transformation of the *q*-values,
- favor the covariates to a degree determined by the data itself,
- result in centered *l*_*j*_, with mean equal to 1, to ease the interpretation,
- let all *l*_*j*_=1 if the external data does not carry any information with predictive value, meaning that the external data is excluded and we end up with the classical penalized methods.

The next two sections explain in detail the construction of such a function in the two cases, adaptive group-regularized ridge regression [13], and our extended version of the weighted lasso with data integration.

### Adaptive group-regularized ridge regression

The Bayesian formulation of ridge regression considers a prior for the *β*_*j*_’s where they are independent and Gaussian distributed with mean zero and variance *τ*^2^. Then the penalty parameter *λ* is proportional to the inverse of this variance. In the adaptive group-regularized ridge regression, first presented in [13], the covariates are divided into groups, and group penalties are introduced, such that all coefficients belonging to a group *g* have the same Gaussian prior with variance $\tau _{g}^{2}$. In our case, the covariates were grouped based on the mentioned *q*-values from the “global test”, such that the covariates with the lowest adjusted *p*-values, the favored covariates, will be in group 1 and so on. We chose the group sizes, such that they reflect the interpretation of the *q*-values [13]. In total, there were 100 groups, where the first group had size 10 (the most relevant covariates), and the following groups had increasing group size, as shown in Additional file 3: Figure S6. The last group (with the highest *q*-values) was the largest group.

The variance for each group *g*, $ \tau ^{2}_{g}$, is estimated by an empirical Bayes approach. The group-specific penalty multiplier for the covariates within group *g* is then $l_{g} = K / \hat { \tau }^{2}_{g}$, where *K* calibrates such that the mean of the inverse penalty multipliers were equal to 1. To enforce monotonicity, the penalty multipliers were calibrated using weighted isotonic regression [26], such that *l*_1_≤…≤*l*_*G*_. After including the penalty multipliers and estimating the resulting updated $\hat {\boldsymbol {\beta }}$, the procedure was iterated by calculating new empirical Bayes estimates, until the cross-validated sum of squares was not improving. This method is implemented in the R package *GRridge* [13].

### Extensions to the weighted lasso with data integration

The weighted lasso with data integration in [16], was presented with a penalty on the form *λ*_*j*_=*λl*_*j*_=*λh*(*η*_*j*_)=*λ*|*η*_*j*_|^*α*^, where *η*_*j*_ was some measure of importance calculated from additional data, and where *α*≥0 controlled the range of the penalty multipliers. The larger the *α* the more difference between favored covariates and unfavored ones. In [16], the optimal combination of *λ* and *α* was found using a 2-dimensional 10-fold cross validation (CV). This procedure could be time consuming, and the two parameters are influenced by each other and potentially unstable. Compared to our approach they did not consider *q*-values as an input.

**Fig. 2:**
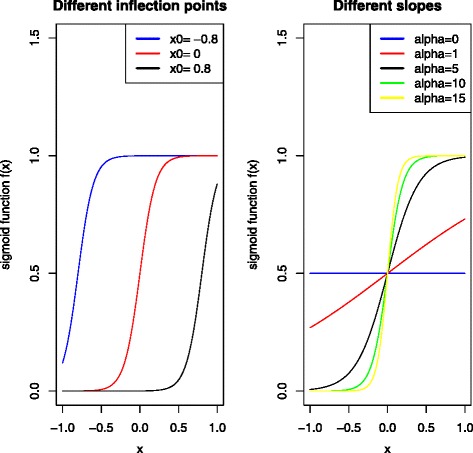
Sigmoid function. A sketch of the sigmoid function $f(x) = 1/\left (1+ e^{-\alpha \left (x - x_{0}\right)}\right)$. In the left panel there are three different values of the inflection point *x*_0_, using *α*=10 in all three cases. In the right panel there are five different values of *α*, using inflection point *x*_0_=0 in all three cases

In our modified version, we used a mapping with a sigmoid shape

$$S(x, \alpha) = \frac{1}{1+ e^{- \alpha (x - x_{0})} }, \quad \text{for }x \in (-1, 1), $$

with *q*-values as input, linearly transformed such that they range from -1 to 1, where *x*_0_ is the inflection point, and *α* again defines the range of the penalty multipliers. See Fig. 2 for different examples of *x*_0_ and *α*. Please note that the argument *x* here is not to be confused with the covariates *x*_*ij*_. This mapping reflects the interpretation of the *q*-values: the smallest *q*-value will be less penalized, and after some significance threshold the penalties increase to a higher level where they stabilize. A natural choice for inflection point is to relate it to FDR equal to 10%. For the method of [16], one needs to carefully select the *η*_*j*_ terms since they affect the model fitting directly. In our case, the sigmoid shape is more robust, since it smooths the *q*-values and is therefore more stable for extreme outliers. Another advantage is that one can also use a priority list as input, then in terms of ranks (1,2,…).

Next, to find the *λ*_*j*_, we calibrated all *l*_*j*_ to have mean equal to one, and used the optimal *λ* found from classical lasso. Then the overall penalty in the weighted penalties case is the same as in the classical case, which has two benefits. First we do not need a 2-dimensional CV, since *α* alone is optimized using CV with the previously calculated *λ* found from classical lasso. Secondly, it eases the interpretation since all *l*_*j*_<1 are favored compared to the classical penalized regression, and vice versa. The resulting function for each penalty multiplier becomes

3 $$\begin{array}{@{}rcl@{}} l_{j} &= K \times S\left(q_{j}^{\ast}, \alpha\right), \end{array} $$

where $q_{j}^{\ast } = 2q_{j} - 1$, the *q*-values linearly transformed to the interval from -1 to 1, and *K* is the normalization constant. We used the R package glmnet [27], which has penalty weights as argument and performs the calculations as explained in Additional file 2: Additional meterials.

### Functional enrichment analysis

To analyze our results from the lasso, we used the Core Expression Analysis function in the Ingenuity Pathway Analysis software (IPA, QIAGEN Redwood City, www.qiagen.com/ingenuity) to identify overrepresented pathways, upstream regulators or diseases in our dataset. The available molecules and relationships in the IPA Knowledge Base for mammal (humans, mouse or rat) were considered, filtering to relationships experimentally observed, directly or indirectly. *P*-values were calculated using the standard right-tailed Fisher exact test within IPA as part of the analysis.

## Results

### Results from the multivariate regression while integrating the associations between DNA-methylation and global gene expression

We quantify the relationship between global bone gene expression and global bone DNA-methylation by looking at two different scenarios. First we considered the association between each DNA-methylation and all transcripts. Next, we considered the association between each DNA methylation and only their cis transcripts, as described in the section “DNA-methylation and cis transcripts”. For the first scenario, the distribution of raw *p*-values from the “global test” is shown in Additional file 4: Figure S7. There are 3830 DNA-methylations significantly associated with global transcripts at FDR =1*%*. Using these multiple test corrected *p*-values, the *q*-values, we fitted the adaptive group-regularized ridge regression, to get an initial impression of the importance of including the external data. To find a shortlist of the most important variables, we then fitted our version of the weighted lasso with data integration. We validated the methods using predictive mean square error (pMSE), by leave one out CV, which in turn leaves one sample out when training the models, and then runs the fitted model on each test sample left out.

### Adaptive group-regularized ridge regression

The adaptive group-regularized ridge regression allows for one penalty per group of covariates. The DNA-methylations were therefore divided into 100 groups based on the *q*-values, representing their strength of association with the gene transcripts. Using these groups, we found the resulting group penalty multipliers (see section “Adaptive group-regularized ridge regression”), and the updated $\hat {\boldsymbol {\beta }}$. The method was then repeated, and converged after three iterations, where the current $\hat {\boldsymbol {\beta }}$ did not improve in terms of cross validated sum of squares.

**Fig. 3:**
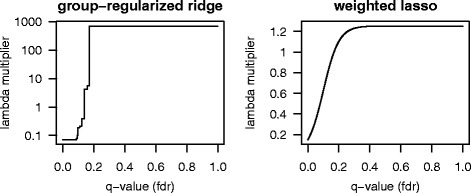
The mapping from *q*-values to penalty multipliers. In the left panel the results from adaptive group-regularized ridge regression are plotted, and in the right panel the results from our version of weighted lasso with data integration are plotted. In both panels the x-axis shows the FDR adjusted *p*-values from the global test, after testing for association between DNA-methylation and at least one gene transcript genome wide. The y-axis shows the corresponding lambda multipliers *l*_*j*_ for each of the methods

The resulting monotonically increasing group penalty multipliers are shown in the left panel in Fig. 3. The 74 first groups (which corresponded to the 32487 DNA-methylations with the lowest *q*-values) were estimated to have equal and lowest penalty weight 0.071. For the consecutive groups the penalty multipliers increased to higher values, and after 13 groups (in total 46266 DNA-methylations) the penalty multipliers stabilized at the highest value 706.73. The last 14 groups (in total 142113 DNA-methylations) got equally high penalty. The highest penalty was relatively large meaning that, in practice, the data showed that these DNA-methylations could be excluded from the model.

After validating the performance, we saw an improvement in pMSE from 2.070 (classical ridge regression) to 1.766 (adaptive group-regularized ridge regression), which was a 14.7*%* improvement. This means that the strength of association between each DNA-methylation and gene transcripts gave important information.

### The extended weighted lasso with data integration

Next, we fitted the extended weighted lasso with data integration and obtained a shortlist of the most important DNA-methylations, which both explained BMD and were correlated to gene expression.

We calculated the optimal *λ* from the classical lasso, using 10-fold cross validation. Next the penalty multipliers *l*_*j*_, described in Eq. (3), were calculated for a grid of *α* values from 0 to 50 in steps of 5. For each *α* the 10-fold cross validated sum of squares error was determined. The optimal *α*, which gave the lowest cross validated error, became 10. The resulting number of variables selected where 22. In the validation step, our version of the weighted lasso gave then a pMSR =2.184, which is an improvement compared to the classical lasso which gave a pMSR =2.630. This corresponded to a 17% improvement.

### Consensus in results from ridge regression and lasso

**Fig. 4:**
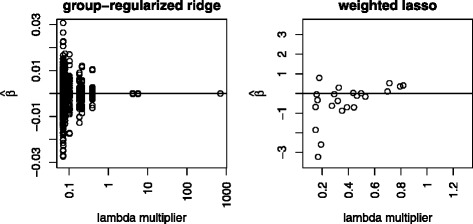
The relationship between the lambda multipliers and the fitted regression coefficients. Distribution of the penalty multipliers versus the resulting $\hat {\boldsymbol {\beta }}$ from adaptive group-regularized ridge regression (left) and our weighted lasso with data integration (right)

Adaptive group-regularized ridge regression and our version of weighted lasso with data integration both improved the predictive error after including the information given by gene expression. The shape of the penalty multipliers, shown in Fig. 3, shows the impact of the information given by the *q*-values from the “global test”. A straight horizontal line would have implied no information in the *q*-values, which was clearly not the case here. We found the same type of shape for the two methods. The Spearman correlation between the penalty multipliers from the two methods was as high as 0.85. In the adaptive group-regularized ridge regression, the only restriction on the shape was monotonicity. Most of the significant *q*-values were indeed favored. Our weighted lasso with data integration, was by definition a sigmoid curve with inflection point placed at the *q*-value =0.1. This variable selection method also showed that the *q*-values do carry information. Figure 4 shows the correspondence between $\hat {\beta }_{j}$ and *l*_*j*_, for all *j*. The largest coefficients had small penalty, as expected. But there were some estimated coefficients with higher penalties that were still strongly present in the final model. This showed that both the individual penalties and the predictive importance (the size of initial $\hat {\boldsymbol {\beta }}$) played a role in the fitting of both methods. Looking at both these different approaches strengthens our impression of the importance of including gene transcripts in the analysis through penalty multipliers.

### The 22 DNA-methylation sites and the calculated effect on BMD

In our weighted lasso with data integration, which performed variable selection, 22 DNA-methylations were selected. The 22 selected DNA-methylations corresponded to 22 different genes. In Additional file 5: Table S1 we see those selected DNA-methylations, the corresponding regression coefficient, and information about the localization of the CpGs. The degree of variation in BMD explained by these 22 DNA-methylations, was calculated by an ordinary least squares regression, using BMD as response and all the 22 DNA-methylations as covariates, resulting in a *R*^2^=0.726. Such a large number of DNA-methylations can possibly explain a large proportion of BMD just by chance, so we compared it to the *R*^2^ for 10000 randomly selected groups of DNA-methylations with group size 22. On average, the random groups had *R*^2^=0.438, clearly lower than the 22 DNA-methylations selected by the lasso regression. None of the random groups had *R*^2^ as large as 0.726.

Additional file 6: Table S2 shows that these DNA-methylations are involved in different biological functions. In this list are transcription factors (ZNF529, NFIA, TFEC, SPI1, MKL1), which have general and probably unique roles in most cells. They represent a link to other genes on the list involved in metabolism (Aldehyde dehydrogenase) that are already strongly implicated as highly associated with BMD [17]. In addition, Destrin, Protocadherin 9 and Dysferlin are known to be important for osteoblasts and/or osteocytes, which are bone anabolic or stress sensing cells, respectively [28–31]. Maybe most notable, 7 genes are specially linked to osteoblast functions and BMD, or generate a bone phenotype when aberrantly expressed (ADAMTS2, COL11A2, TFEC, DOCK5, PTPN11, ANXA2, DSTN). The results from the Ingenuity Pathway Analysis in Additional file 7: Table S3, show that several general functions and pathways might be affected by DNA-methylation in these 22 genes, which are discussed in the last part of the next section.

### DNA-methylation and cis transcripts

As mentioned earlier, we also looked at the association between each DNA-methylation site and their cis gene expression. Additional file 8: Figure S8 shows the resulting *p*-values from the “global test” when testing each methylation against only their cis related transcripts. There were 981 methylations with significant association at a FDR=1*%*. Using the adjusted *p*-values from these type of “global tests”, we saw little or no improvement in the predictive performance. For the adaptive group-regularized ridge regression we got a pMSE =2.04, which was a small improvement compared to the classical Ridge with pMSE=2.07. When fitting our weighted lasso with data integration, the optimal *α* became 0 such that all *l*_*j*_=1, which means that the external data did not improve the prediction.

## Discussion

### Summary of the statistical approach

The present paper studies the association between bone mineral density (BMD) and global DNA-methylation using penalized regression. Since DNA-methylation may affect gene expression, transcripts for the same individuals were integrated into the analysis by use of individual penalties for each DNA-methylation. The DNA-methylations which were strongly associated to global gene expression, got a lower penalty than the DNA-methylations with weaker associations to gene expression. The degree to which these associations lead to differentiated penalties, was determined by the data. The predicted mean square error was thereafter considerably improved.

### Discussing the statistical significance of the 22 CpGs found

In our list of 22 selected CpGs, the most familiar pathway belonging to bone metabolism (e.g. Wnt signaling and TGF *β* signaling) did not come up, and this may be due to the variable selection procedure in lasso. If covariates are strongly correlated, e.g. variables existing in networks, lasso tends to select only one out of several highly correlated variables [14], and the variable selection can therefore result in different short lists [32–34]. Our list of 22 DNA-methylations is the most important one, but there may also be other lists with bordering predictive value.

We investigated the possible difference between cis associations versus global association, and only saw improvements in the predictions after integrating information from global associations. When cis related genes were considered, no (or a small) improvement in the predictions was detected. The lack of improved predictive performance in the cis analysis was somewhat surprising, and may be related to the complex networks behind the machinery of gene expression. Thus, genes further down in the pathways could be more correlated, and therefore the trans-effects will be more visible when using penalty weights. This indicates that strong associations between DNA-methylations and genes at other positions in the genome, could be of importance.

### Robustness of hyper parameters

Our results were robust against the choice of the hyper parameters in the models. For our weighted lasso with data integration, the inflection point in the sigmoid function is a hyper parameter and was chosen based on the biological interpretation, and put equal to 0.1. We also tried other choices, still having the same interpretation as a significance check point, and the results and pMSE were not markedly changed. In the adaptive group-regularized ridge regression different choices of group sizes were applied, but made no notable changes to the predicted mean square error. Additionally, if we randomly permuted our calculated penalty weights, the integrated methods performed similar to the classical regression methods, in other words the external randomized information is not improving the prediction and therefore discarded.

### Discussing our and similar methods

A hot topic of discussion is whether or not to standardize the covariates in penalized regression [35]. If we standardize, the idea of one common penalty is more appropriate, while on the other hand rescaling the data may remove some of the (differential) signal and may lead to instabilities of variables for which the sample variances are small. Standardization is equivalent to introducing a penalty multiplier that is proportional to the variance in the unstandardized setting [35]. So if one chooses to standardize, the type of standardization (the weights) will influence the results, because some covariates may be relatively more favored than others. If the original covariates have similar range and variance, it could be best not to standardize. DNA-methylation levels are relative measures, where all values lie between 0 (no DNA-methylation) and 1 (all methylated). These DNA-methylation levels are therefore already on the same scale and thus comparable, so we have chosen not to standardize.

There are several methods allowing for individual and groupwise penalties. The weights used in adaptive lasso are simply $l_{j} = 1/\hat {\beta }_{j}^{L}$, where $\hat {\beta }_{j}^{L}$ is the estimate from the classical lasso, and *λ* is again selected by cross validation [22]. Here, the covariates, which lasso finds important in the initial run, are given a lower penalty in the second step. Tai and Pan [24], used groups of covariates, and a *λ*_*g*_ for each group *g* is found by cross validation, for *g*=1,…,*G*. This means that a G-dimensional cross validation is needed. For large *G* this is computationally too demanding to solve. In that case, they suggested to use *λ*_*g*_=*λl*_*g*_, and let *l*_*g*_ be the group mean of the original, not shrunken coefficients, similar to adaptive lasso. To our knowledge, [16] was the first to introduce weights based on external data. If the external data set possesses important information, the data integrated weights will strengthen the prediction, regardless of the results from the classical lasso. In [23], they build upon this and use *p*-values corrected for multiple testing as a basis. But in that paper they focus on dividing the covariates into two groups, where the first group should not be subject to selection and are given weights small enough to ensure their inclusion in the model. An alternative to the mentioned group penalty methods is the group lasso [36], where instead the external data is used to group the covariates, and a ridge penalty is forced on the groups.

### Biological relevance of the 22 selected CpG sites in relation to bone

The human genome DNA methylation pattern is changing throughout life, from conception to old age [37, 38]. These changes preserve our epigenetic heritage and are important for e.g. regulating tissue differentiation [39]. The present material offers a unique opportunity to study the correlation between DNA methylation and gene expression associated with a common phenotype with strong genetic disposition - BMD. In addition, we extract differentially methylated CpGs between healthy compared to osteoporotic postmenopausal women by doing a robust genome-wide analysis. To document specific functions related to the 22 bone associated methylations discovered in this study, will require experiments performed in bone cell cultures and/or in gene modified animals. We present below their functional assignment in bone as it is known from the literature.

Using Ingenuity Pathway Analysis software, we identified the top canonical pathways related to our list of CpG sites, shown in Additional file 7: Table S3. The most familiar pathways in relation to bone metabolism (like the Wnt or TGFB signaling pathways) are not among the significant results in IPA. However, osteoimmunology is now an established field and bone metabolism has been shown to be affected, e.g. via CD28 receptor signaling on T-cells, which in turn affects Wnt signaling in bone cells [40]. Thus, identification of “CD28 Signaling in T Helper Cells” as the top canonical pathway may indicate that this pathway is more important for bone metabolism than previously understood. The top ranked upstream regulator ERG (ETS Transcription Factor) may act on BMD via regulation of mesenchymal cell differentiation in cooperation with TGF- *β* [41]. It is also worth noting that we have identified miR-16-5p, second ranked among upstream regulators, to be among the mature bone miRNAs most significantly correlated to BMD (unpublished). Cancer appeared as top ranked among related diseases and disorders. Uncontrolled cell growth may involve a vast variety of epigenetic aberrations resulting in e.g. alterations of intracellular signaling related to a number of different diseases, probably also affecting bone metabolism. Hematological and Immunological Disease ranked second and third, respectively, reflecting the importance of blood/immune cells in bone metabolism as mentioned above.

## Conclusions

Overall, in our version of the weighted lasso with data integration and the adaptive group-regularized ridge regression, the key advantages are that both methods allow for ordered penalty terms and thus a tentative ranking of the covariates or groups of covariates. Both methods let the data decide upon the range of the weights and thus the relevance of the external data, by either an additional parameter *α* or by iterations. In addition, the penalty parameter is defined by *λ*_*j*_=*λl*_*j*_ where *λ* is fixed to be the optimal value from the classical penalized regression without integration, which makes the computations faster and more stable. Improved predictive performance was found for both methods after integrating gene expression data. We also conclude that the presented selection method, the weighted lasso with data integration, is successful in detecting DNA-methylations that are related to BMD, verifying genes that are known to be bone related, and moreover, detecting novel transcripts of potential importance for bone metabolism and osteoporosis. The overall impact from this paper is how adaptation and improved integrative methodology, enable us to delineate the association between DNA-methylation and gene expression in the analysis of BMD.

## Additional files

Additional file 1 **Figure S5.** Infinium HumanMethylation450 BeadChip provides a broad coverage throughout gene regions, as well as CpG islands, shelves and shores, as graphically visualized in this reprint from [42]. Abbreviations; TSS: transcription start site. TSS1500: 200–1500 bases upstream of the TSS. TSS200: 0-200 bases upstream of the TSS. UTR: untranslated region. 5’UTR: Within the 5’ untranslated region, between the TSS and the ATG start site. Body: Between the ATG and stop codon; irrespective of the presence of introns, exons, TSS, or promoters. 3’UTR: Between the stop codon and poly A signal. A CpG island is based on UCSC criteria: CG content > 50%, length > 200 bps, and a ratio > 0.6 of observed number of CpG dinucleotides to the expected number. Shore: 0–2 kb from island. Shelf: 2–4 kb from island. N: upstream (5’) of CpG island. S: downstream (3’) of CpG island. (PNG 218 kb)

Additional file 2 **Additional materials.** Section 1: More on optimizing individual penalties in penalized regression. Section 2: More on the “global test” [20]. (PDF 131 kb)

Additional file 3 **Figure S6.** The groups sizes in adaptive group-regularized ridge regression. The DNA-methylation sites were divided into 100 groups based on the *q*-values from the global test (as explained in “Quantifying the strength of association between DNA-methylation and gene expression” section), where group one is the group with the smallest *q*-values. The number of DNA-methylation sites in the first group is 10, and then the group sizes increases more and more. Group number 100 is the largest group with the highest *q*-values. (PDF 5 kb)

Additional file 4 **Figure S7.** Distribution of *p*-values. The distribution of *p*-values from the “global test”, when testing for association between each DNA-methylation against all transcripts. (PDF 9 kb)

Additional file 5**Table S1.** The 22 identified CpGs selected by our weighted Lasso with data integration, together with their annotation. The first columns give the corresponding gene symbol and Illumina ID. Next follows the estimated regression coefficient $\hat {\boldsymbol {\beta }} $ from the optimal fitted model. The locations are described by CHR (chromosome) and Mapinfo (base pair position on the chromosome). The annotations are described by Probe SNPs (single nucleotide polymorphisms) Probe SNPs 10 (one SNP within 10 base pairs of the CpG site), Ucsc refgene group (see Additional file 1: Figure S5 for more details). Relation to ucsc CpG island (where the label Island is a region with high frequency of CpG sites, N Shore or S Shore are neighboring region 5’ or 3’ to CpG island, N Shelf or S Shelf are neighboring region 5’ or 3’ to CpG shore), DMR (differentially methylated regions), Enhancer (region of DNA that binds proteins to regulate transcription of a gene), Regulatory feature group (Promoter Associated or unclassified) and DHS (DNase I hypersensitive sites are regions where the chromatin has lost its condensed structure, exposing the DNA and making it extra accessible to DNAse cleavage). No mark means no available information. Information is taken from Illumina. (XLSX 48 kb)

Additional file 6 **Table S2.** The 22 identified CpGs selected by our weighted Lasso with data integration, together with information about their function and relation to bone. The functions are found using the web-site www.genecards.org, unless referred to by citation. No mark means no relevant information. (XLSX 34 kb)

Additional file 7 **Table S3.** Ingenuity Pathway Analysis. Summary from Ingenuity Pathway Analysis (www.ingenuity.com) on the 22 selected methylation sites from our weighted Lasso with data integration. (XLSX 40 kb)

Additional file 8 **Figure S8.** Distribution of *p*-values. The distribution of *p*-values from the “global test”, when testing for association between each DNA-methylation against the cis related transcripts (meaning that the DNA-methylation and transcript are from the same gene). (PDF 9 kb)
