# Supplementary figures and images for: Integrated analysis of DNA-methylation and gene expression using high-dimensional penalized regression: a cohort study on bone mineral density in postmenopausal women
Source: BMC Med Genomics. 2018 Mar 7;11:24. doi: 10.1186/s12920-018-0341-2 (PMC5842543; doi:10.1186/s12920-018-0341-2)

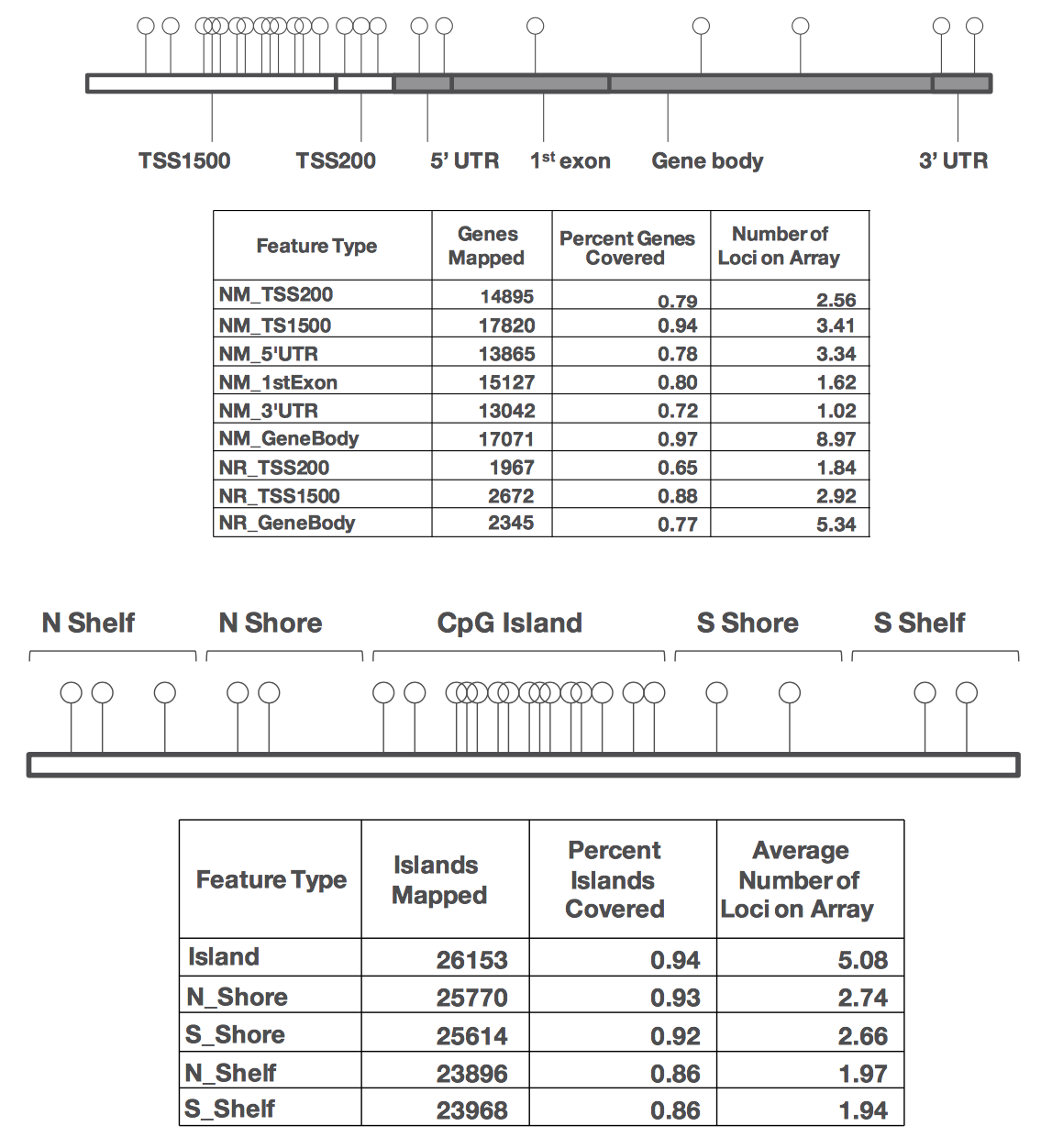

Supplement: Supplementary file 1 — Figure S5. Infinium HumanMethylation450 BeadChip provides a broad coverage throughout gene regions, as well as CpG islands, shelves and shores, as graphically visualized in this reprint from [42]. Abbreviations; TSS: transcription start site. TSS1500: 200–1500 bases upstream of the TSS. TSS200: 0-200 bases upstream of the TSS. UTR: untranslated region. 5’UTR: Within the 5’ untranslated region, between the TSS and the ATG start site. Body: Between the ATG and stop codon; irrespective of the presence of introns, exons, TSS, or promoters. 3’UTR: Between the stop codon and poly A signal. A CpG island is based on UCSC criteria: CG content > 50%, length > 200 bps, and a ratio > 0.6 of observed number of CpG dinucleotides to the expected number. Shore: 0–2 kb from island. Shelf: 2–4 kb from island. N: upstream (5’) of CpG island. S: downstream (3’) of CpG island. (PNG 218 kb) [file 12920_2018_341_MOESM1_ESM.png]

# Adaptive group-regularized ridge regression

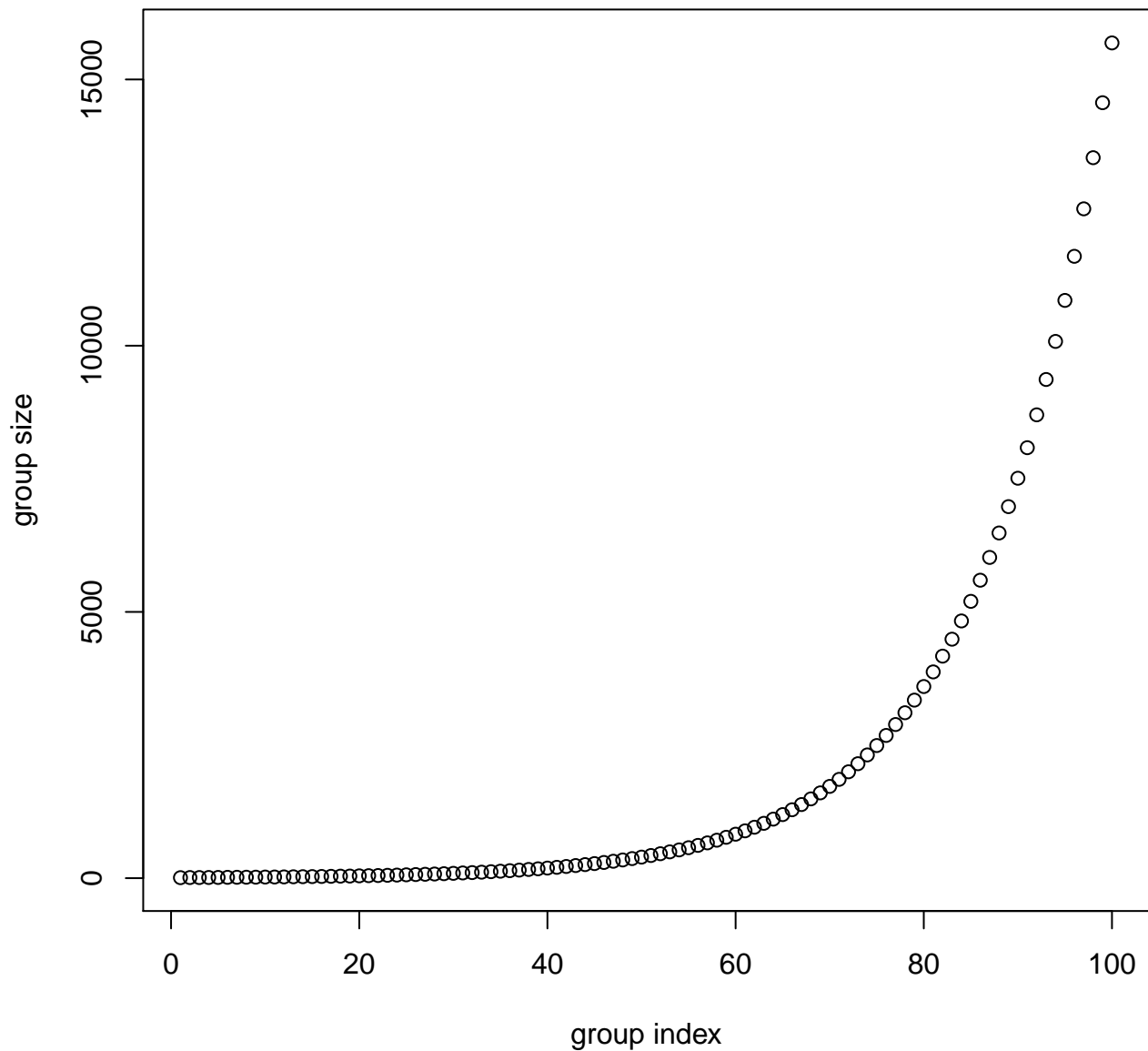

Supplement: Supplementary file 3 — Figure S6. The groups sizes in adaptive group-regularized ridge regression. The DNA-methylation sites were divided into 100 groups based on the q-values from the global test (as explained in “Quantifying the strength of association between DNA-methylation and gene expression” section), where group one is the group with the smallest q-values. The number of DNA-methylation sites in the first group is 10, and then the group sizes increases more and more. Group number 100 is the largest group with the highest q-values. (PDF 5 kb) [file 12920_2018_341_MOESM3_ESM.pdf]

## All transcripts

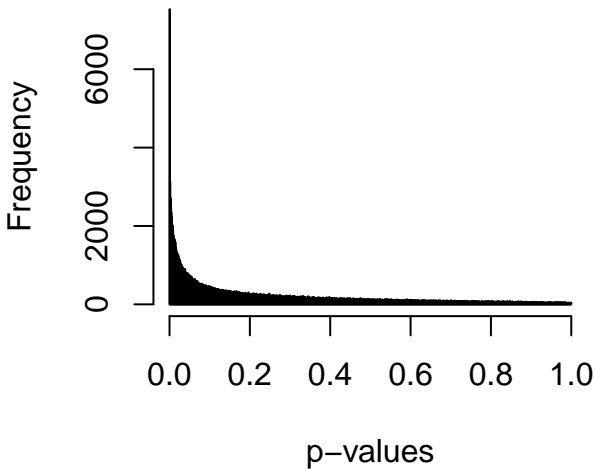

Supplement: Supplementary file 4 — Figure S7. Distribution of p-values. The distribution of p-values from the “global test”, when testing for association between each DNA-methylation against all transcripts. (PDF 9 kb) [file 12920_2018_341_MOESM4_ESM.pdf]

## Cis transcripts

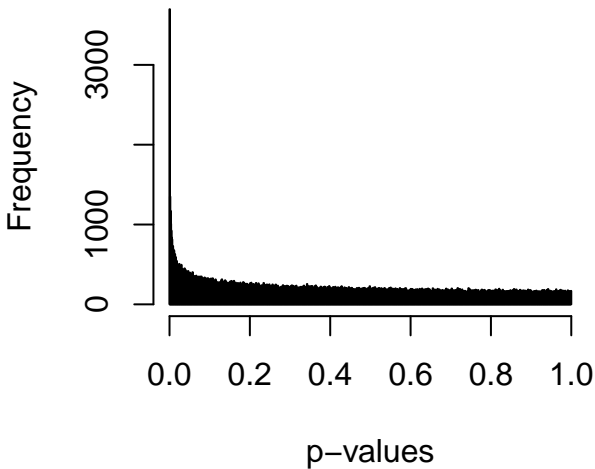

Supplement: Supplementary file 8 — Figure S8. Distribution of p-values. The distribution of p-values from the “global test”, when testing for association between each DNA-methylation against the cis related transcripts (meaning that the DNA-methylation and transcript are from the same gene). (PDF 9 kb) [file 12920_2018_341_MOESM8_ESM.pdf]
