# Supplementary material for: Integrated analysis of DNA-methylation and gene expression using high-dimensional penalized regression: a cohort study on bone mineral density in postmenopausal women
Source: BMC Med Genomics. 2018 Mar 7;11:24. doi: 10.1186/s12920-018-0341-2 (PMC5842543; doi:10.1186/s12920-018-0341-2)
Supplement: Supplementary file 2 — Additional materials. Section 1: More on optimizing individual penalties in penalized regression. Section 2: More on the “global test” [20]. (PDF 131 kb) [file 12920_2018_341_MOESM2_ESM.pdf]

Supplementary material

Integrated analysis of DNA-methylation and gene expression using high-dimensional penalized regression:

a cohort study on bone mineral density in postmenopausal women.

Tonje G Lien[1*], Ørnulf Borgan[1], Sjur Reppe[2,3], Kaare Gautvik[3,4], Ingrid Kristine Glad[1]

[1] University of Oslo, Department of Mathematics, Oslo, Norway. [2] Oslo University Hospital, Department of Medical Biochemistry, Oslo, Norway. [3] Lovisenberg Diakonale Hospital, Unger-Vetlesen Institute, Oslo, Norway. [4] University of Oslo, Institute of Basic Medical Sciences, Oslo, Norway.

[*] Correspondence: tonje.lien@rr-research.no [1] University of Oslo, Department of Mathematics, Oslo, Norway.

Additional material 1 — More on optimizing individual penalties in penalized regression

In section 1.3 Penalized regression using penalty multipliers, we looked at the optimization scheme for fitting a high dimensional linear regression model, by using penalized sum of squares

$$\sum_{i=1}^{80}(y_i - \boldsymbol{x}_i^T\boldsymbol{\beta})^2 + \sum_{j=1}^{p}\lambda_j J(\beta_j),$$

where we included individual penalty terms λ_j. This optimization procedure can, after some scaling of the data, be rewritten as the classical case with one common λ. This scaling can be done also for generalized linear models, by using the log likelihood. For normally distributed data the same optimal solution can be found from minimizing penalized sum of squares or by maximizing the penalized log-likelihood $l(\boldsymbol{\beta}|\boldsymbol{y},\boldsymbol{X}) - \sum_{j=1}^{p}\lambda_j J(\beta_j)$. We therefore illustrate the scaling from individual penalties to one common penalty by using the log-likelihood first with the lasso, next with ridge regression.

For individual i, let y_i be the observed response and $\boldsymbol{x}_i = (x_{i1}, \ldots, x_{ip})^T$ the observed covariates, where $i = 1, \ldots, n$. Let $\boldsymbol{y} = (y_1, \ldots, y_n)$ and $\mathbf{X}$ be the $n \times p$ matrix with rows $\mathbf{x}_i^T$. Remember that for generalized linear models, the log likelihood is $l(\boldsymbol{\beta}|\boldsymbol{y},\boldsymbol{X}) = \log \Pi_i f(y_i|\mu_i)$ where $g(\mu_i) = \boldsymbol{x}_i^T\boldsymbol{\beta}$, for a link function $g()$ using coefficients $\boldsymbol{\beta} = (\beta_1, \ldots, \beta_p)^T$. If we, for some $l_j > 0$, scale each column j of $\boldsymbol{X}$ by $1/l_j$ to get a new design matrix $\boldsymbol{X}^*$, and at the same time scale β_j by l_j getting a new coefficient vector $\boldsymbol{\beta}^*$, we do not change the expression for the log likelihood, thus $l(\boldsymbol{\beta}|\boldsymbol{y},\boldsymbol{X}) = l(\boldsymbol{\beta}^*|\boldsymbol{y},\boldsymbol{X}^*)$. Then we write

$$l(\boldsymbol{\beta}|\boldsymbol{y},\boldsymbol{X}) - \lambda\textstyle\sum_j l_j|\beta_j| = l(\boldsymbol{\beta}^*|\boldsymbol{y},\boldsymbol{X}^*) - \lambda\sum_j|\beta_j^*|,$$

and find the estimate of $\boldsymbol{\beta}^*$ using standard procedures. The individual penalty estimate is $\hat{\beta}_j = \hat{\beta}_j^*/l_j$. For ridge regression, we do the same type of rescaling, only that we use $\sqrt{l_j}$ and find

$$l(\boldsymbol{\beta}|\boldsymbol{y},\boldsymbol{X}) - \lambda\sum_j l_j\beta_j^2 = l(\boldsymbol{\beta}|\boldsymbol{y},\boldsymbol{X}) - \lambda\sum_j(\sqrt{l_j}\beta_j)^2 = l(\boldsymbol{\beta}^*|\boldsymbol{y},\boldsymbol{X}^*) - \lambda\sum_j\beta_j^{*2}.$$

Again, the individual penalty estimates are found by transforming back, $\hat{\beta}_j = \hat{\beta}_j^*/\sqrt{l_j}$.

Additional material 2 — More on the "global test"

When testing each DNA-methylation site M_j against all gene expressions $G_1, \ldots, G_p$ from the same individuals, we have a high dimensional alternative, and the global test was used [1]. Suppose that the DNA methylation M_j has a distribution dependent on the p variables $G_1, \ldots, G_p$ through some coefficients $\boldsymbol{\gamma} = (\gamma_1, \ldots, \gamma_p)$. Then the test is formulated as $H_0 : \boldsymbol{\gamma} = 0$ against $H_A : \boldsymbol{\gamma} \neq 0$. The null hypothesis is that there is no association between DNA-methylation site M_j and the p variables, and the alternative hypothesis that one or more variable is associated with M_j. A prior distribution is assumed for $\boldsymbol{\gamma}$, where $Cov(\gamma) = \tau^2\Sigma$, and the test can be written as $H_0 : \tau^2 = 0$ against $H_A : \tau^2 > 0$. For the linear model, they showed that the test has good power, even in low dimensions [1].

References

[1] Goeman, J.J., Van De Geer, S.A., Van Houwelingen, H.C.: Testing against a high dimensional alternative. Journal of the Royal Statistical Society: Series B **68**(3), 477–493 (2006)
